# Evaluation of the Interactions between Water Extractable Soil Organic Matter and Metal Cations (Cu(II), Eu(III)) Using Excitation-Emission Matrix Combined with Parallel Factor Analysis

**DOI:** 10.3390/ijms160714464

**Published:** 2015-06-25

**Authors:** Jing Wei, Lu Han, Jing Song, Mengfang Chen

**Affiliations:** 1 Key Laboratory of Coastal Environmental Processes and Ecological Remediation, Yantai Institute of Coastal Zone Research (YIC), Chinese Academy of Sciences (CAS), Shandong Provincial Key Laboratory of Coastal Environmental Processes, YICCAS, 17 Chunhui Road, Yantai 264003, Shandong, China; E-Mail: jwei@yic.ac.cn; 2 Key Laboratory of Soil Environment and Pollution Remediation, Institute of Soil Science, Chinese Academy of Sciences, 71 East Beijing Road, Nanjing 210008, Jiangsu, China; E-Mails: hanlu@issas.ac.cn (L.H.); jingsong@issas.ac.cn (J.S.)

**Keywords:** water extractable organic matter, copper, europium, excitation-emission matrix, parallel factor analysis

## Abstract

The objectives of this study were to evaluate the binding behavior of Cu(II) and Eu(III) with water extractable organic matter (WEOM) in soil, and assess the competitive effect of the cations. Excitation-emission matrix (EEM) fluorescence spectrometry was used in combination with parallel factor analysis (PARAFAC) to obtain four WEOM components: fulvic-like, humic-like, microbial degraded humic-like, and protein-like substances. Fluorescence titration experiments were performed to obtain the binding parameters of PARAFAC-derived components with Cu(II) and Eu(III). The conditional complexation stability constants (log*K*_M_) of Cu(II) with the four components ranged from 5.49 to 5.94, and the Eu(III) log*K*_M_ values were between 5.26 to 5.81. The component-specific binding parameters obtained from competitive binding experiments revealed that Cu(II) and Eu(III) competed for the same binding sites on the WEOM components. These results would help understand the molecular binding mechanisms of Cu(II) and Eu(III) with WEOM in soil environment.

## 1. Introduction

Dissolved organic matter (DOM) is ubiquitous in soil system. Although being small in weight percentages, DOM is the most labile and reactive fraction of the multicomponent soil organic matter pool. As DOM plays a significant role in the biogeochemical cycling of trace metals, remarkable efforts have been made to classify the processes involved in trace metal interacting with DOM, through both experimental and modeling approaches [1,2,3]. Nevertheless, the processes are not fully understood. For example, determination of metal binding constants with DOM is still hampered, due to the intrinsic complexity of DOM, the lack of stoichiometric information, and analytical limitations [4]. Trace metals complexation with DOM remains poorly defined at the molecular scale under relevant environmental conditions, such as low concentrations of metals relative to DOM [5].

Excitation-emission matrix combined with parallel factor analysis (EEM-PARAFAC) is a selective, high sensitive and non-destructive technique. The EEM fluorescence spectra can be seen as an overall “fingerprint” of DOM bulk samples that include the key components [6]. However, the overlapping of the fluorescence spectra between complex DOM components may lead to confusion in subsequent data analysis. PARAFAC analysis, a multiway data analysis method, can model the complex EEM landscape and extract chemically meaningful spectral and concentration components [7,8]. Moreover, this combined technique is applicable to low DOM (less than 10 mg/L) and metal ion concentrations of environmental relevance. The EEM-PARAFAC derived soil DOM components mainly included humic- and fulvic-, and protein-like substances [9,10,11]. The humic and fulvic components, which carry a large number of functional groups, can play a dominant role in interacting with metals in terrestrial environments [12]. EEM-PARAFAC has been applied to study the interaction between metals such as Al(III) and Fe(III) with soil DOM components [10,13].

Dissolved organic matter in soil samples, is often extracted with water or dilute aqueous salt solutions in laboratory experiments [14,15]. In this paper, we will emphasize the interaction of metal cations (Cu(II) and Eu(III)) with water extractable organic matter (WEOM) from soil. Cu is a plant nutrient at low concentrations but harmful at high concentrations [16]. Increased application of wastewater, sludge and manure helped Cu accumulation in agricultural soils worldwide [17,18,19]. Eu(III) is often used as an analogue for trivalent lanthanium, a member of the rare earth elements (REEs). REEs may reach soil system through mining production [20], application of REEs-enriched fertilizers [21] and animal manure containing the lanthanides [22]. As an emerging pollutant, the accumulation of REEs in soil, their bioaccumulation in crops, and entry to the food chain are of growing ecological and human health concerns in China [23,24,25].

In order to gain a better understanding of the behavior of the REEs and heavy metals in the soil environment, detailed investigation is warranted to identify the mechanisms of REEs interacting with soil WEOM, with the existence of competing heavy metal cations. Therefore, the primary objectives of the present study were: (1) To identify the main binding components in soil WEOM and evaluate the binding constants of each component for Cu(II) and Eu(III) using EEM combined with PARAFAC analysis; and (2) To explore the binding sites and the competing effect of Cu(II) and Eu(III) with soil WEOM.

## 2. Results and Discussion

### 2.1. Fluorescence Properties of Water Extractable Organic Matter (WEOM)

**Figure 1:**
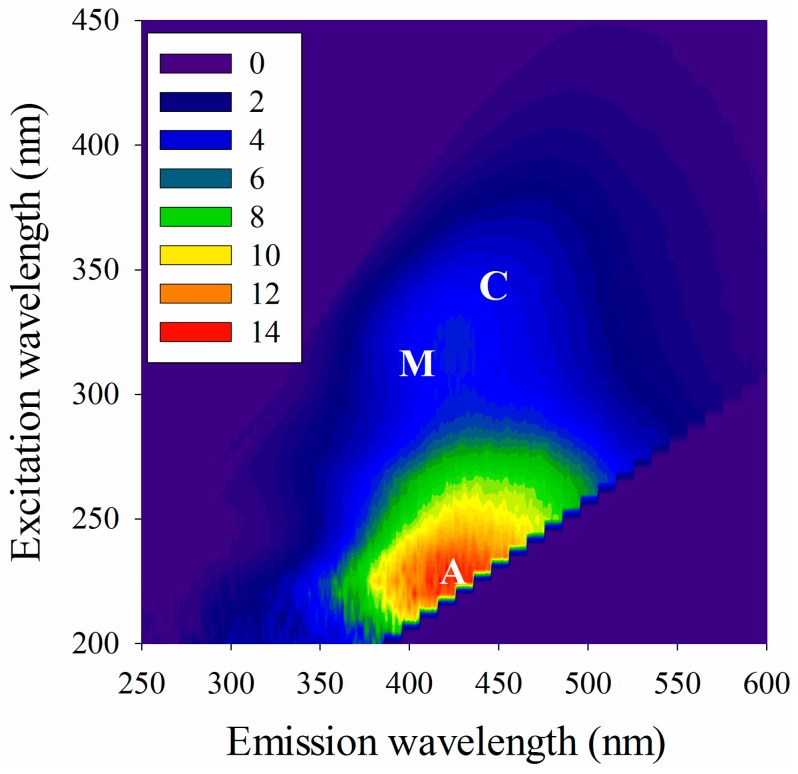
The fluorescence excitation-emission matrix (EEM) spectrum of a water extractable organic matter (WEOM) sample without metal titration.

An example is shown in Figure 1 the fluorescence EEM spectrum of a diluted WEOM sample without metal titration. Visual inspection of the WEOM spectrum suggests the presence of three fluorophores at Excitation/Emission (*E*x/*E*m) wavelength pairs of 230/440, 330/400, and 350/450 nm. The 230/440 and 350/450 nm peaks, presented in the ultraviolet range and in the visible range, were designated as A and C, respectively, and were attributed to humic-like substances [26]. The 330/400 nm peak presented in the ultraviolet range was designated as a microbial degraded humic (M) peak [9].

### 2.2. Parallel Factor Analysis (PARAFAC) Derived Components

**Figure 2:**
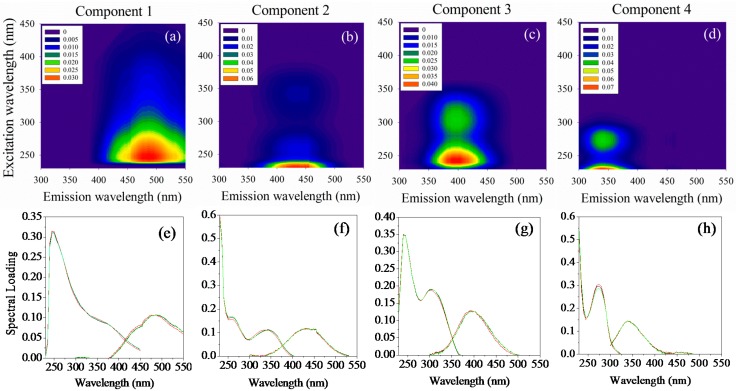
The contour plots show the *E*x/*E*m locations of PARAFAC-derived components (**a–d**); The line plots show the spectral loadings of four components modeled with the halves of the dataset (green and red lines) and on the whole dataset (black lines) (**e–h**).

Four fluorescent components were identified by PARAFAC analysis based on the split-half validation procedure (Figure 2). The largely overlapping excitation and emission loadings of the four components, modeled with the halves of the dataset and on the whole dataset are also shown in Figure 2. All fluorescent components had single emission maximum and single or multiple excitation maxima. Component 1 (*E*x/*E*m = 250/460) was classified as fulvic-like substance, and component 2 (*E*x/*E*m = (235, 330)/440) was designated as humic-like substances. Component 3 (*E*x/*E*m = (240, 300)/400) was ascribed as microbial degraded humic-like substances. Component 4 (*E*x/*E*m = (<235, 275)/340) was protein-like, containing mainly tryptophan-like substances. In the case of the high fluorescence intensity of the fulvic and humic peaks masking the protein peak presented in the bulk sample spectra, the PARAFAC can decompose the complex mixture effectively. These components had been previously identified in the terrestrial environment [27,28].

The fluorescence intensity per unit TOC [QSU/(mg/L)] of components 1–4 were 0.74, 1.39, 0.56 and 0.27, and their relative abundance to total fluorescence were 25.1%, 46.8%, 19.0% and 9.1%, respectively. Fulvic-like and humic-like components are most commonly abundant in terrestrial DOM, such as DOM from soil extractions, soil solution, wetlands, and forested streams. Components derived from PARAFAC in previous studies that fall within the microbial degraded humic-like component were variable in occurrence and abundance. However, it was found that the identity of this component increased following biodegradation of plant biomass and manure [28,29,30].

### 2.3. Interaction of Fluorescent Components with Cu(II) and Eu(III)

**Figure 3:**
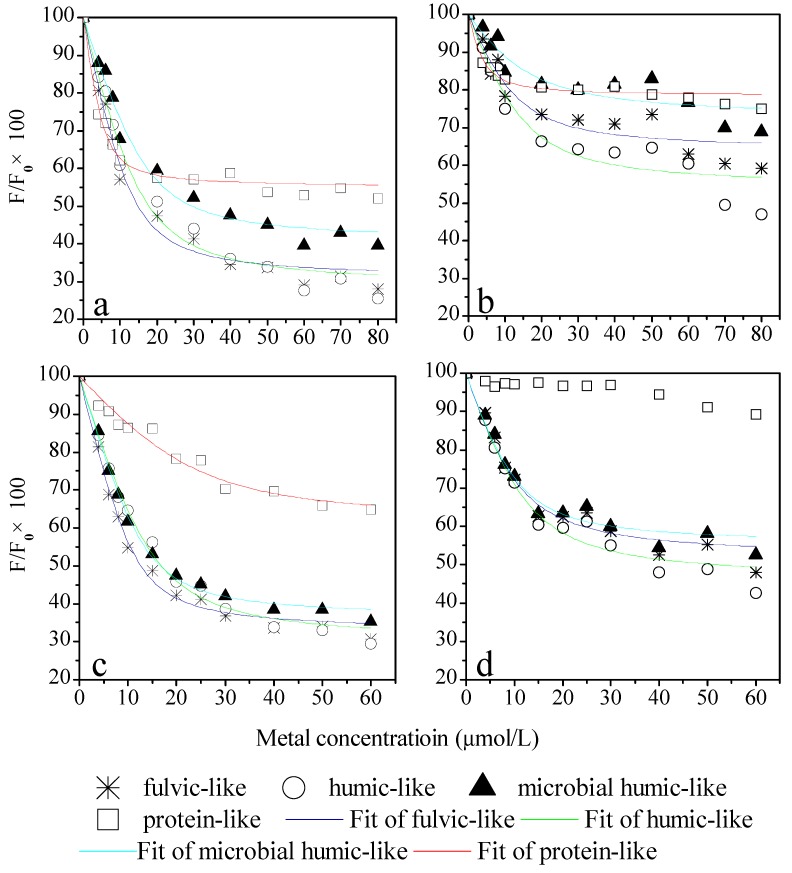
The fluorescence quenching curves of PARAFAC-derived components with Cu (0–80 μmol/L) (**a**); Cu (0–80 μmol/L) and Eu (20 μmol/L) (**b**); Eu (0–60 μmol/L) (**c**); Eu (0–60 μmol/L) and Cu (10 μmol/L) (**d**).

The fluorescence quenching curves of PARAFAC-derived components with Cu(II) and Eu(III) are shown in Figure 3. As initial levels of fluorescence intensity were different among four components, the fluorescence quenching curves were shown as percent changes from initial levels (*F*/*F*_0_ × 100, where *F* and *F*_0_ are the fluorescence intensity with and without titrated metals, respectively).

Fluorescence quenching occurred for all the PARAFAC derived components investigated in this study, indicating the occurrence of electronic structural changes in the WEOM components by forming complexes with Cu(II) and Eu(III). Along with the addition of metal cations, the fluorescence identities of four components firstly decreased sharply in a low range of metal concentration (0–10 μmol/L), and then decrease slowly in the high range of metal concentration (20–80 μmol/L) (Figure 3a,c). However, the quenching effects of specific components with Cu(II) and Eu(III) were different. Table 1 summarizes the conditional binding parameters of PARAFAC derived components with both cations. The conditional complexation stability constants (log*K*_M_) of Cu(II) for WEOM components are close to those reported by other investigators using fluorescence quenching titration (FQT). For instances, the Cu(II) complexation stability constants were reported ranging from log*K*_M_ of 4.65 to 5.55 for the soil- and compost-borne humic acid [31,32]. The Cu(II) log*K*_M_ for the fulvic-like component and microbial humic-like component, which derived from surface water-borne DOM, were reported ranging from 4.26 to 4.69, and 4.20 to 6.76, respectively [33]. Previous studies mostly focused on the binding capacity of Eu(III) with specifically extracted fulvic acid and humic acid. The Eu(III) log*K*_M_ were reported ranging between 4.90 to 5.03 for soil fulvic acid, 5.98 to 6.09 for soil humic acid, and 5.61 to 5.71 for Aldrich humic acid [34]. This study is a step forward in that Eu(III) stability constants with four components of soil WEOM were derived simultaneously (Table 1).

**Table 1:** Conditional binding parameters of PARAFAC-derived components with Cu(II) and Eu(III) determined by modified Ryan and Weber Model.

PARAFAC Component	*C*_M_ (μmol/L)	log *K*_M_	*C*_L_ (μmol/L)	*f* (%)	*R*^2^
fulvic-like	*C*_Cu_ of 0 to 80	5.66	12.7	69.3	0.982
humic-like		5.49	14.8	71.6	0.978
microbial humic-like		5.53	16.8	59.6	0.981
protein-like		5.94	5.35	45.0	0.975
fulvic-like	*C*_Cu_ of 0 to 80 with *C*_Eu_ of 20	5.40	11.4	36.2	0.891
humic-like		5.38	13.7	45.9	0.910
microbial humic-like		5.15	12.9	27.5	0.840
protein-like		5.77	3.31	21.7	0.937
fulvic-like	*C*_Eu_ of 0 to 60	5.81	10.9	67.2	0.990
humic-like		5.58	14.5	70.3	0.990
microbial humic-like		5.76	13.1	63.8	0.994
protein-like		5.26	22.8	38.7	0.964
fulvic-like	*C*_Eu_ of 0 to 60 with *C*_Cu_ of 10	5.47	9.15	48.3	0.962
humic-like		5.48	11.4	54.1	0.968
microbial humic-like		5.58	9.67	44.9	0.966
protein-like		Not modeled			

In the case of complexation with the fulvic-, humic-like, and microbial degraded humic-like components, the log*K*_M_ values for Eu(III) were larger than those for Cu(II). On the contrary, the log*K*_M_ values for Eu(III) with protein were smaller than that for Cu(II). These results indicate that, within the entire range of metal concentrations in this study, Eu(III) had a stronger binding capability than Cu(II) with the fulvic-, humic-like, and microbial degraded humic-like components, but not with protein-like component. The log*K*_M_ values for Cu(II) with four components were in the order: protein-like > fulvic-like > microbial degraded humic-like > humic-like component, and for Eu(III) the order was fulvic-like > microbial degraded humic-like > humic-like component > protein-like. Besides, the fraction of the initial fluorescence that corresponds to the binding fluorophores (*f*) and the stoichiometric concentration of the ligand (*C*_L_) values varied between four components interacting with Cu(II) and Eu(III). These results suggested that the binding processes varied with cations and WEOM components.

For Cu(II) and Eu(III), binding occurs mostly with the carboxylic and phenolic groups of the natural organic matter [35]. Luster *et al.* [36] have investigated the binding of Cu(II) to leaf litter extracted DOM using fluorescence spectrometry and electron spin resonance spectroscopy. The results showed that stable inner-sphere complexes of Cu(II) and the DOM could be assigned mainly to binding sites formed by carboxylic and/or phenolic O ligand atoms, and the weak Cu(II) binding may be caused by out-sphere complexes involving ketonic, phenolic and carboxylic groups. Fourier transform infrared spectra responses on Cu(II) with DOM revealed that carboxylic groups were most likely the dominant binding site involved in complexation [37,38]. The important roles played by carboxylic and phenolic groups in forming Eu(III) complexes of humic and fulvic aicd were confirmed using various spectroscopic method [39,40]. Thus, Cu(II) and Eu(III) may bind with WEOM components through its carboxylic and phenolic groups. Compared with humic acid, fulvic acid contains a larger number of carboxylic sites and relatively few phenolic sites [41]. The various types and amounts of functional groups in fulvic acid and humic acid substances might cause the differences in cation binding behavior between the two components. Extended study is needed to evidence the above hypothesis. It should also be noted that the nonfluorescent substances (*i.e.*, polysaccharides, lipids and lignin) in WEOM can interact with cations. Further study is needed to look into the binding strength and sequencing of functional groups with cations, such as using two-dimensional FTIR correlation spectroscopy [10].

It is well-known that fluorescence of protein-like substances is quenched or enhanced due to interaction with metal cations. However, there were few studies using the FQT to characterize the heavy metal binding capacity of soil protein-like substances. When charactering the interaction between surface water-borne DOM with heavy metals, it was found that the utility of FQT combining synchronous and EEM spectra might be limited, because the fluorescent peaks of protein-like substances in bulk DOM EEM were often overlapped with those of fulvic- and humic-like peaks [33,42,43]. In our study, the FQT combining EEM PARAFAC allowed evaluating the binding potential of Cu(II) and Eu(III) with specific soil protein-like components. The degree of quenching protein-like component decreased rapidly at low metal concentration (0–10 μmol/L), while reached steady soon compared with those of the other three components. It was reported that the fluorescence intensity of surface water-borne protein decreased at the earlier stage of Cu(II) addition, but sharply increased with further increasing of Cu(II) [33]. The exact reason for these findings is not yet clear, but it may be related to the different sensitivity of protein-like substances to different metals and changes in molecular environment of proteins.

### 2.4. Competitive Reactions between Cu(II) and Eu(III) with WEOM

Data from competition experiments between metal cations with WEOM may help understand the behaviors of metal cations in soil systems. At the beginning of the competitive experiment the WEOM was loaded with Eu(III). After stepwise addition of the Cu(II), the quenching effects of four components with Cu(II) (Figure 3b) were all less pronounced than the controls (Figure 3a). Furthermore, the log*K*_M_, *f*, and *C*_L_ values for Cu(II) with four components all decreased (Table 1). Similarly, the quenching effects and corresponding binding parameters of fulvic-, humic- and microbial degraded humic-like components with Eu(III) reduced after loaded with Cu(II). The quenching curve of protein-like component in Figure 3d was not modeled because of the poor fitness with testing data. Our results suggest that the binding sites within each WEOM component for both cations are probably the same. This is in agreement with Marang *et al.* [44] who evaluated the Eu(III) binding behavior with humic acid in the presence of competitive Cu(II). Their results demonstrated that the strong Cu(II) competitive binding led to increased concentration of free Eu(III) cation in solution, and the competitive binding sites were mainly carboxylic and phenolic groups. Similarly, Konstantinou *et al.* [35] showed that after stepwise addition of the Eu(III), Cu(II) adsorbed on the olive cake-derived DOM were partly replaced. The conditional complexation stability constants of the Cu(II)-DOM and Eu(III)-DOM complexes they evaluated were 5.3 and 6.3 at pH 6, respectively.

The relatively strong interaction of Cu(II) and Eu(III) cations to the soil WEOM could have significant implications on the bioavailability and mobility of these and homologue trace metal cations in natural environment, especially when the application of sludge and manure increased amount of soil WEOM. The trace metals pre-bound to soil WEOM could be replaced by competing metals, and then released into soil solution as free metal cations, becoming bioavailable. Additionally, the mobility of these trace metals can be enhanced due to their strong binding to the WEOM. Further experiments and model calculations are still needed to better understand the competition of Cu(II) and Eu(III) cations with functional group-sites of WEOM, and their influences on Cu(II) and Eu(III) behaviors in the soil system.

## 3. Experimental Section

### 3.1. Soil Sampling and WEOM Extraction

Soils were collected from Nanjing, Jiangsu province, China (31°41ʹ N, 118°52ʹ E). The soil was classified as Ferri-Udic Argosols. Soil samples (0–20 cm) were collected using a 5 cm internal diameter auger from 12 random cores collected in three plots. The fresh soil was mixed thoroughly, air-dried, and sieved through 2 and 0.25 mm screens for further analysis [9]. The soil organic matter (SOM) content of the mixed sample was 32.36 ± 0.78 g/kg (mean ± standard deviation). The WEOM was extracted with deionized water (Milli-Q, Millipore, Billerica, MA, USA) (1:5 *w*/*v* soil-to-water ratio) by shaking air-dried soil samples (<2 mm sieved) for 24 h on a horizontal shaker at 25 °C. The WEOM solution was filtered using 0.45 μm polytetrafluoroethylene (PTFE) filters and stored at 4 °C for analysis.

### 3.2. Fluorescence Quenching Titration

Copper and Europium stock solutions (0.1 and 1.0 mol/L) were prepared using Cu(NO_3_)_2_ and Eu(NO_3_)_3_ (Aladdin, Shanghai, China). Prior to fluorescence titration, the WEOM were diluted with Milli-Q water to TOC < 10 mg/L to minimize inner-filtration effects. Aliquots of 30 mL of the diluted WEOM were titrated with stock solutions in 50 mL brown sealed vials using an automatic syringe. The Cu(II) concentrations in the final solutions ranged from 0 to 80 μmol/L (0, 4, 6, 8, 10, 20, 30, 40, 50, 60, 70 and 80 μmol/L), and the Eu(III) concentrations ranged from 0 to 60 μmol/L (0, 4, 6, 8, 10, 15, 20, 25, 30, 40, 50 and 60 μmol/L). Experiments with higher Eu(III) concentrations were not performed because they would be irrelevant for most natural conditions. To maintain constant pH before and after titration, the titrated solutions were adjusted to pH 6.0 using HNO_3_ or NaOH (0.1 and 1.0 mol/L). All titrated solutions were shaken for 24 h at 25 °C to ensure complexation equilibrium. Then, all titrated solutions were analyzed by fluorescence excitation-emission matrix (EEM) spectroscopy (Hitachi High Technologies, Tokyo, Japan).

### 3.3. Competitive Reactions

Competition reactions between Cu(II) and Eu(III) with WEOM were carried out by batch experiments in brown sealed vials by adding stock solution containing one of the metals to a solution containing constant amount WEOM (TOC < 10 mg/L) and the other metal. Solutions of diluted WEOM loaded with Eu(III) 20 μmol/L were titrated with Cu(II) in the concentration ranged from 0 to 80 μmol/L (0, 4, 6, 8, 10, 20, 30, 40, 50, 60, 70 and 80 μmol/L). Similarly, solutions of diluted WEOM loaded with Cu(II) 10 μmol/L was titrated with Eu(III) in the concentration ranged from 0 to 60 μmol/L (0, 4, 6, 8, 10, 15, 20, 25, 30, 40, 50 and 60 μmol/L). The titrated solutions were adjusted to pH 6.0 using HNO_3_ or NaOH (0.1 and 1.0 mol/L). After shaken 24 h for equilibration at 25 °C, the titrated solutions were analyzed by fluorescence EEM spectroscopy.

### 3.4. Fluorescence EEM Determination and PARAFAC Modeling

The fluorescence EEMs were measured on a Hitachi F-7000 fluorescence spectrometer (Hitachi High Technologies, Tokyo, Japan) in scan mode. Scanning emission (*E*m) spectra from 250 to 600 nm were obtained in 1 nm increments by varying the excitation (*E*x) wavelength from 200 to 450 nm in 5 nm increments. The spectra were recorded at a scan rate of 2400 nm/min, using excitation and emission slit band widths of 5 nm.

Water Raman scatter peaks were eliminated by subtracting a Milli-Q water blank from the EEMs. The Rayleigh scattering effects were removed [45]. Then, EEMs were calibrated and normalized in quinine sulfate units (QSU), where 1 QSU is the maximum fluorescence intensity of 0.01 mg/L of quinine (qs) in 1 N H_2_SO_4_ at the *E*x/*E*m = 350/450.

The PARAFAC analysis was performed using the DOMFluor toolbox (www.models.life.ku.dk) for MATLAB R2011a [46]. We deleted excitation wavelengths from 200 to 235 nm, and emission wavelengths from 250 to 300 nm and from 550 to 600 nm. A total of 48 EEMs were applied for the PARAFAC analysis. The PARAFAC models with two to six components were computed for the EEMs. Determination of the correct number of components was primarily based on split half analysis, residual analysis, and visual inspection [46].

### 3.5. Complexation Modeling

For this study, the modified Ryan–Weber model [31,47] was applied to estimate the binding parameters between the PARAFAC derived components and metals (Cu(II) and Eu(III)). The major assumptions of this model are that 1:1 metal-ligand complexes are formed and that a linear relationship exists between the metal-bound ligand concentration and the quenched WEOM fluorescence intensity. Since PARAFAC can decompose the complex mixture of WEOM fluorophores into independent fluorescence components, the application of this model may provide more appropriate information than the fluorescence intensity from the peak maxima of EEM spectra.

The modified Ryan–Weber model equation is given by:

(1) $$\frac{F}{F_{0}}=1+(\frac{F_{\text{ML}}}{F_{0}}-1)\;(\frac{1}{2K_{\text{M}}C_{\text{L}}})\times [1+K_{\text{M}}C_{\text{L}}+K_{\text{M}}C_{\text{M}}–\sqrt{{\left(1+\;K_{\text{M}}C_{\text{L}}+K_{\text{M}}C_{\text{M}}\right)}^{2}-4K_{M}{}^{2}C_{L}C_{M}}]$$

where *F*, and *F*_0_ are the measured fluorescence intensities of a specific fluorophore at the metal concentration *C*_M_ and at the beginning of the titration (in the absence of titrated metals), respectively. *F*_ML_ is the limiting fluorescence intensity below which the intensity does not change upon the metal addition (*i.e.*, metal-saturated complex). *K*_M_ and *C*_L_ are the conditional stability constant and the stoichiometric concentration of the ligand, respectively. Using nonlinear regression analysis to estimate three parameters in Equation (1) could lead to unreasonably small values for *C*_L_ [36]. The number of fitting parameters of Equation (1) was reduced using the modified Ryan and Weber model [36].

(2) $$\left|\frac{F}{F_{0}}\;-1\right|=\left|\frac{F_{\text{ML}}}{F_{0}}\;-1\right|\times \left(1-e^{-\text{α}C_{\text{M}}}\right)$$

where *F*_ML_/*F*_0_ − 1 and α are the fitting parameters. ORIGIN software (Version 8.0, OriginLab, Northampton, MA, USA) was used to solve for *F*_ML_, *K*_M_, and *C*_L_. In addition, the fraction of the initial fluorescence that corresponds to a specific binding fluorophore (*f*) was determined using Equation (3) [33].

(3) $$f=\frac{\left(F_{0}-F_{\text{ML}}\right)}{F_{0}}\;\times 100\%$$

## 4. Conclusions

Application of EEM-PARAFAC divided soil WEOM into four components, namely fulvic-, humic-, microbial degraded humic-, and protein-like components. The main benefit of the EEM-PARAFAC to this study was to obtain quantitative information on the binding capacity of heterogeneous WEOM fluorescent components with Cu(II) and Eu(III) at molecular level. Compared with Cu(II), Eu(III) presents a higher affinity for the fulvic-, humic-like, and microbial humic-like components, but not for protein-like component. Cu(II) and Eu(III) may compete for the same binding sites of the WEOM components.

## References

[1] Marsac R., Davranche M., Gruau G., Dia A., Pédrot M., le Coz-Bouhnik M., Briant N. (2013). Effects of Fe competition on REE binding to humic acid: Origin of REE pattern variability in organic waters. Chem. Geol..

[2] Tipping E. (1998). Humic ion-binding model VI: An improved description of the interactions of protons and metal ions with humic substances. Aquat. Geochem..

[3] Milne C.J., Kinniburgh D.G., van Riemsdijk W.H., Tipping E. (2003). Generic NICA-donnan model parameters for metal-ion binding by humic substances. Environ. Sci. Technol..

[4] Gasper J.D., Aiken G.R., Ryan J.N. (2007). A critical review of three methods used for the measurement of mercury (Hg^2+^)-dissolved organic matter stability constants. Appl. Geochem..

[5] Aiken G.R., Hsu-Kim H., Ryan J.N. (2011). Influence of dissolved organic matter on the environmental fate of metals, nanoparticles, and colloids. Environ. Sci. Technol..

[6] Li W.H., Sheng G.P., Liu X.W., Yu H.Q. (2008). Characterizing the extracellular and intracellular fluorescent products of activated sludge in a sequencing batch reactor. Water Res..

[7] Bro R. (1997). PARAFAC. Tutorial and applications. Chemom. Intell. Lab. Syst..

[8] Andersen C.M., Bro R. (2003). Practical aspects of PARAFAC modeling of fluorescence excitation-emission data. J. Chemom..

[9] Erich M.S., Plante A.F., Fernández J.M., Mallory E.B., Ohno T. (2012). Effects of profile depth and management on the composition of labile and total soil organic matter. Soil Sci. Soc. Am. J..

[10] Yu G.H., Wu M.J., Wei G.R., Luo Y.H., Ran W., Wang B.R., Zhang J.C., Shen Q.R. (2012). Binding of organic ligands with Al(III) in dissolved organic matter from soil: Implications for soil organic carbon storage. Environ. Sci. Technol..

[11] Ishii S.K.L., Boyer T.H. (2012). Behavior of reoccurring PARAFAC components in fluorescent dissolved organic matter in natural and engineered systems: A critical review. Environ. Sci. Technol..

[12] Ahmed I.A.M., Taylor J.H., Bieroza M., Zhang H., Davison W. (2014). Improving and testing geochemical speciation predictions of metal ions in natural waters. Water Res..

[13] Ohno T., Amirbahman A., Bro R. (2008). Parallel factor analysis of excitation-emission matrix fluorescence spectra of water soluble soil organic matter as basis for the determination of conditional metal binding parameters. Environ. Sci. Technol..

[14] Bolan N.S., Adriano D.C., Kunhikrishnan A., James T., McDowell R., Senesi N. (2011). Dissolved organic matter: Biogeochemistry, dynamics, and environmental significance in soils. Adv. Agron..

[15] Guigue J., Mathieu O., Leveque J., Mounier S., Laffont R., Maron P.A., Navarro N., Chateau C., Amiotte-Suchet P., Lucas Y. (2014). A comparison of extraction procedures for water-extractable organic matter in soils. Eur. J. Soil Sci..

[16] Gaetke L. (2003). Copper toxicity, oxidative stress, and antioxidant nutrients. Toxicology.

[17] Epstein L., Bassein S. (2001). Pesticide applications of copper on perennial crops in California, 1993 to 1998. J. Environ. Qual..

[18] Bolan N., Adriano D., Mahimairaja S. (2004). Distribution and bioavailability of trace elements in livestock and poultry manure by-products. Crit. Rev. Environ. Sci. Technol..

[19] Heijerick D.G., van Sprang P.A., van Hyfte A.D. (2006). Ambient copper concentrations in agricultural and natural european soils: An overview. Environ. Toxicol. Chem..

[20] Chen Z. (2011). Global rare earth resources and scenarios of future rare earth industry. J. Rare Earths.

[21] Hu Z., Richter H., Sparovek G., Schnug E. (2004). Physiological and biochemical effects of rare earth elements on plants and their agricultural significance: A review. J. Plant Nutr..

[22] He M.L., Wehr U., Rambeck W.A. (2010). Effect of low doses of dietary rare earth elements on growth performance of broilers. J. Anim. Physiol. Anim. Nutr..

[23] Wang L.H., Huang X.H., Zhou Q. (2008). Effects of rare earth elements on the distribution of mineral elements and heavy metals in horseradish. Chemosphere.

[24] Guo W., Zhao R.X., Zhao W.J., Fu R.Y., Guo J.Y., Bi N., Zhang J. (2013). Effects of arbuscular mycorrhizal fungi on maize (*Zea mays* L.) and sorghum (*Sorghum bicolor* L. Moench) grown in rare earth elements of mine tailings. Appl. Soil Ecol..

[25] Brioschi L., Steinmann M., Lucot E., Pierret M.C., Stille P., Prunier J., Badot P.M. (2013). Transfer of rare earth elements (REE) from natural soil to plant systems: Implications for the environmental availability of anthropogenic REE. Plant Soil.

[26] Coble P.G. (1996). Characterization of marine and terrestrial dom in seawater using excitation emission matrix spectroscopy. Mar. Chem..

[27] Chen M., Price R.M., Yamashita Y., Jaffé R. (2010). Comparative study of dissolved organic matter from groundwater and surface water in the florida coastal everglades using multi-dimensional spectrofluorometry combined with multivariate statistics. Appl. Geochem..

[28] Hunt J.F., Ohno T. (2007). Characterization of fresh and decomposed dissolved organic matter using excitation-emission matrix fluorescence spectroscopy and multiway analysis. J. Agric. Food Chem..

[29] Zhang Y., van Dijk M.A., Liu M., Zhu G., Qin B. (2009). The contribution of phytoplankton degradation to chromophoric dissolved organic matter (CDOM) in eutrophic shallow lakes: Field and experimental evidence. Water Res..

[30] Beggs K.M., Summers R.S. (2011). Character and chlorine reactivity of dissolved organic matter from a mountain pine beetle impacted watershed. Environ. Sci. Technol..

[31] Plaza C., Brunetti G., Senesi N., Polo A. (2006). Molecular and quantitative analysis of metal ion binding to humic acids from sewage sludge and sludge-amended soils by fluorescence spectroscopy. Environ. Sci. Technol..

[32] Hernandez D., Plaza C., Senesi N., Polo A. (2006). Detection of copper(II) and zinc(II) binding to humic acids from pig slurry and amended soils by fluorescence spectroscopy. Environ. Pollut..

[33] Yamashita Y., Jaffe R. (2008). Characterizing the interactions between trace metals and dissolved organic matter using excitation-emission matrix and parallel factor analysis. Environ. Sci. Technol..

[34] Terashima M., Nagao S., Iwatsuki T., Fujitake N., Seida Y., Iijima K., Yoshikawa H. (2012). Europium-binding abilities of dissolved humic substances isolated from deep groundwater in Horonobe area, Hokkaido, Japan. J. Nucl. Sci. Technol..

[35] Konstantinou M., Kolokassidou K., Pashalidis I. (2009). Studies on the interaction of olive cake and its hydrophylic extracts with polyvalent metal ions (Cu(II), Eu(III)) in aqueous solutions. J. Hazard. Mater..

[36] Luster J., Lloyd T., Sposito G., Fry I.V. (1996). Multi-wavelength molecular fluorescence spectrometry for quantitative characterization of copper(II) and aluminum(III) complexation by dissolved organic matter. Environ. Sci. Technol..

[37] Evangelou V.P., Marsi M., Chappell M.A. (2002). Potentiometric-spectroscopic evaluation of metal-ion complexes by humic fractions extracted from corn tissue. Spectrochim. Acta A.

[38] Pan B., Qiu M., Wu M., Zhang D., Peng H., Wu D., Xing B. (2012). The opposite impacts of Cu and Mg cations on dissolved organic matter-ofloxacin interaction. Environ. Pollut..

[39] Shin H.S., Rhee S.W., Lee B.H., Moon C.H. (1996). Metal binding sites and partial structures of soil fulvic and humic acids compared: Aided by Eu(III) luminescence spectroscopy and DEPT/QUAT ^13^C NMR pulse techniques. Org. Geochem..

[40] Lukman S., Saito T., Aoyagi N., Kimura T., Nagasaki S. (2012). Speciation of Eu^3+^ bound to humic substances by time-resolved laser fluorescence spectroscopy (TRLFS) and parallel factor analysis (PARAFAC). Geochim. Cosmochim. Acta.

[41] Milne C.J., Kinniburgh D.G., Tipping E. (2001). Generic NICA-donnan model parameters for proton binding by humic substances. Environ. Sci. Technol..

[42] Fu P., Wu F., Liu C., Wang F., Li W., Yue L., Guo Q. (2007). Fluorescence characterization of dissolved organic matter in an urban river and its complexation with Hg(II). Appl. Geochem..

[43] Maie N., Scully N.M., Pisani O., Jaffe R. (2007). Composition of a protein-like fluorophore of dissolved organic matter in coastal wetland and estuarine ecosystems. Water Res..

[44] Marang L., Reiller P.E., Eidner S., Kumke M.U., Benedetti M.F. (2008). Combining spectroscopic and potentiometric approaches to characterize competitive binding to humic substances. Environ. Sci. Technol..

[45] Zhang Y., Liu X., Wang M., Qin B. (2013). Compositional differences of chromophoric dissolved organic matter derived from phytoplankton and macrophytes. Org. Geochem..

[46] Stedmon C.A., Bro R. (2008). Characterizing dissolved organic matter fluorescence with parallel factor analysis: A tutorial. Limnol. Oceanogr-Meth..

[47] Ryan D.K., Weber J.H. (1982). Fluorescence quenching titration for determination of complexing capacities and stability-constants of fulvic-acid. Anal. Chem..

